# Transforming growth factor-β1 impairs neuropathic pain through pleiotropic effects

**DOI:** 10.1186/1744-8069-5-16

**Published:** 2009-03-27

**Authors:** Stefania Echeverry, Xiang Qun Shi, Alexandra Haw, Hong Liu, Zhong-wei Zhang, Ji Zhang

**Affiliations:** 1 The Alan Edwards Centre for Research on Pain, McGill University, 740, Dr. Penfield Ave. Montreal, Quebec, Canada; 2 The Jackson Laboratory, 600 Main Street, Bar Harbar, ME 04609, USA

## Abstract

**Background:**

Understanding the underlying mechanisms of neuropathic pain caused by damage to the peripheral nervous system remains challenging and could lead to significantly improved therapies. Disturbance of homeostasis not only occurs at the site of injury but also extends to the spinal cord and brain involving various types of cells. Emerging data implicate neuroimmune interaction in the initiation and maintenance of chronic pain hypersensitivity.

**Results:**

In this study, we sought to investigate the effects of TGF-β1, a potent anti-inflammatory cytokine, in alleviating nerve injury-induced neuropathic pain in rats. By using a well established neuropathic pain animal model (partial ligation of the sciatic nerve), we demonstrated that intrathecal infusion of recombinant TGF-β1 significantly attenuated nerve injury-induced neuropathic pain. TGF-β1 treatment not only prevents development of neuropathic pain following nerve injury, but also reverses previously established neuropathic pain conditions. The biological outcomes of TGF-β1 in this context are attributed to its pleiotropic effects. It inhibits peripheral nerve injury-induced spinal microgliosis, spinal microglial and astrocytic activation, and exhibits a powerful neuroprotective effect by preventing the induction of ATF3^+ ^neurons following nerve ligation, consequently reducing the expression of chemokine MCP-1 in damaged neurons. TGF-β1 treatment also suppresses nerve injury-induced inflammatory response in the spinal cord, as revealed by a reduction in cytokine expression.

**Conclusion:**

Our findings revealed that TGF-β1 is effective in the treatment of neuropathic by targeting both neurons and glial cells. We suggest that therapeutic agents such as TGF-β1 having multipotent effects on different types of cells could work in synergy to regain homeostasis in local spinal cord microenvironments, therefore contributing to attenuate neuropathic pain.

## Background

Neuropathic pain caused by primary lesions in the peripheral nerve or by dysfunctions in the central nervous system (CNS) has an enormous negative impact on the quality of life of individuals affected by this condition. Unfortunately, many forms of neuropathic pain cannot be adequately treated using conventional analgesics [1]; they can only be partially managed with antidepressants and antiepileptics, with varying levels of success [2]. Pathogenesis of this hypersensitive state is very complex, involving structural, physiological and pharmacological changes throughout the neuroaxis (from the site of peripheral nerve injury to the spinal cord/brain). While a neuron-centric view has dominated the literature for decades, recent work has uncovered extensive neuroimmune interactions as substrates of neuropathic pain. Interactions between the immune and nervous systems occur at multiple levels, where different types of immune/glial cells and immune-derived substances are implicated in various stages of pathogenesis [3].

Peripheral nerve injury can induce spinal inflammatory reactions in the spinal cord and activation of microglia and astrocytes [4-6]. Nerve injury-induced spinal microglial activation results in activation of preexisting resident microglia, generation of new cells [7] and recruitment of peripheral macrophages [8]. Both resident and bone marrow-derived microglia are involved in the central component of sensitization that enhances neuronal excitability. A correlation between persistent activation of spinal astrocytes and chronic pain has also been established and is a common feature of chronic pain in different animal models following peripheral nerve injury [9], spinal cord injury [10] and bone cancer [11]. As a result of either primary injury to sensory neurons via mechanical insults or as a secondary consequence of apoptotic cell death, damaged neurons release a number of substances such as cytokines, chemokines, excitatory amino acids and ATP, which can in turn trigger surrounding glial activation [12]. An early, transient and robust reaction of microglia is required for initiation of nerve injury-induced hyperalgesia, since they not only phagocytose cellular materials but also produce and secrete pro-inflammatory molecules that evoke an increase in neuronal activity of the spinal cord dorsal horn [13]. Sustained astrocytic reaction in the spinal cord plays an important role in maintaining neuropathic pain [14]. Both activated microglia and astrocytes are key players in the central neuroinflammation process responsible for hyper-excitability of spinal nociceptive neurons at different stages of pathogenesis.

The transforming growth factor (TGF)-β family members are cytokines that fulfil key functions during development and maintain adult tissue homeostasis. To date, three isoforms have been identified in mammalian tissues; TGF-β1, TGF-β2 and TGF-β3 [15]. They act in a highly contextual manner depending on the cell type and microenvironment. TGF-βs may promote cell survival or induce apoptosis, stimulate cell proliferation or induce differentiation, and their immune functions are mostly anti-inflammatory [16]. The biological effects of TGF-βs are transduced through the type I (RI) and type II (RII) transmembrane receptors. TGF-βs signalling involves binding of the ligand to the constitutively active serine/threonine kinase receptor RII and subsequent recruitment of RI into a signalling complex [17]. Downstream signalling is mediated through activation of the Smad family of proteins [17], which translocate into the nucleus to regulate gene transcription [17]. In normal adult animals, TGF-β2 and β3 are ubiquitously expressed in neurons and glia cells in both CNS and PNS, whereas TGF-β1 is restricted to the meninges [18]. However, up-regulation of TGF-β1 has been reported in the brains of animals with neurodegenerative disease and following ischemic injury [19]. Both *in vitro *and *in vivo *studies have illuminated the biological functions of TGF-β1 on neurons and glial cells. TGF-β1 controls proliferation of neurons and by acting together with other trophic factors such as GDNF, regulates neuronal survival [20]. TGF-β1 blocks microglial proliferation and free radical induction [21]. Many of the effects of TGF-β1 on astroglia are anti-inflammatory and immunosuppressive [16]. TGF-β1 inhibits up-regulation of TNF-α induced by interferon-α and IL-1β [22]. TGF-β1-/- mice that died at 3–4 weeks exhibited spontaneously elevated iNOS expression and increased NO creation products [23].

In the present study, we sought to determine whether TGF-β1 can be used to alleviate neuropathic pain caused by nerve injury. We hypothesized that through its multiple effects on different types of cells, TGF-β1 could contribute to restore homeostasis in a spinal cord with direct damage to the peripheral nerve, thereby inhibiting central sensitization and attenuating chronic hypersensitivity. By using a well established model [24] of neuropathic pain, we demonstrated that intrathecal infusion of recombinant TGF-β1 was not only effective in preventing hypersensitivity following nerve injury, but also capable of reversing thermal hyperalgesia and mechanical allodynia. The impact of TGF-β1 on behavioural outcomes was due to its pleiotropic effects on both neurons and glia, including preventing neuronal damage, inhibiting spinal microgliosis, inhibiting spinal microglial and astrocyte activation and reducing central inflammatory response.

## Results

### Intrathecal administration of TGF-β1 significantly attenuated nerve injury-induced hypersensitivity

**Figure 1:**
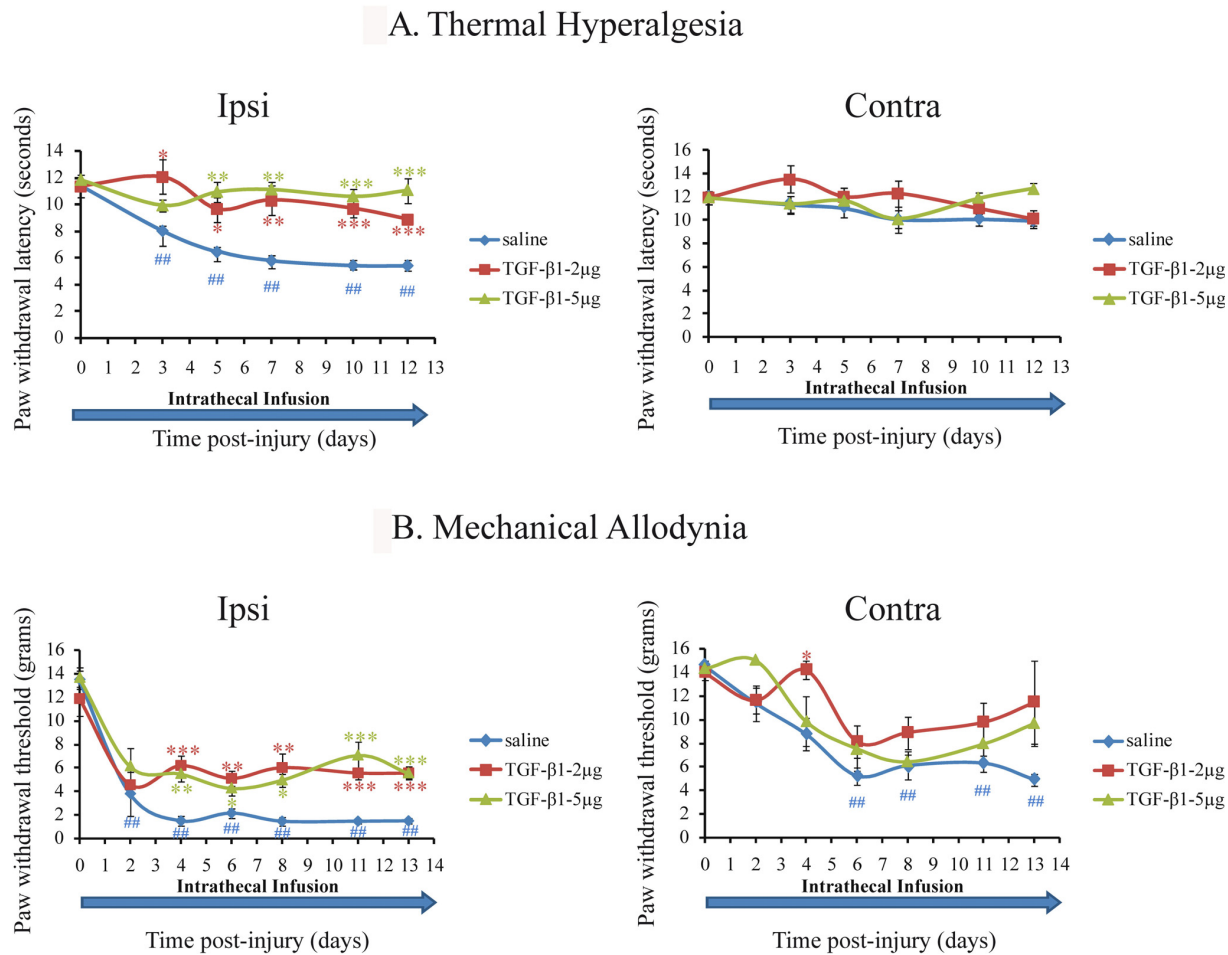
**Intrathecal TGF-β1 infusion prevented the development of mechanical allodynia and thermal hyperalgesia**. Shortly after partial sciatic nerve ligation, rats developed a unilateral hypersensitivity to heat stimuli (**A**) and a sharp bilateral decrease in paw withdrawal threshold in response to Von Frey hair stimulation at the plantar surface (**B**). Note that chronic intrathecal infusion of TGF-β1 (2 μg and 5 μg) started on the day of surgery for 14 days (indicated by the blue arrow) almost completely prevented the initiation of thermal hyperalgesia and partially attenuated mechanical allodynia (**A-B**). Data are shown as means ± SEM. **p < 0.01, ***p < 0.001, TGF-β1 treated vs saline treated at each time point, ##p < 0.01, each time point vs. presurgery baseline (d0) in the group of injured-saline-treated rats, n = 6 per group.

To test the hypothesis that TGF-β1 alleviates behavioural signs of neuropathic pain, we first evaluated the effects of TGF-β1 on the development of mechanical allodynia and thermal hyperalgesia in rats following nerve injury. Rats started to receive TGF-β1 or saline infusion on the day of the surgery and treatment lasted 14 days. As illustrated in Fig 1, shortly after partial ligation of the left sciatic nerve, rats receiving saline infusion showed an exaggerated unilateral response to heat stimuli (Fig. 1A) and a sharp bilateral decrease in response to Von Frey hair stimulation at the plantar surface (Fig. 1B), which confirmed a similar observation reported by Seltzer et al (1990). TGF-β1 did not alter paw withdrawal threshold and latency in animals without injury (data not shown). However, central infusion of TGF-β1 almost completely abolished thermal hyperalgesia 14 days after nerve injury (89% reduction with 5 g TGF-μβ1) (Fig. 1A). Paw withdrawal latency in response to heat stimuli was 11.04 ± 0.93 sec (5 μg TGF-β1) and 8.84 ± 0.18 sec (2 μg TGF-β1) vs. 5.42 ± 0.37 sec in animals receiving saline treatment (P < 0.001 vs. saline treated), whereas pre-surgery paw withdrawal latency was 11.51 ± 0.84 sec. Nerve injury-induced mechanical allodynia was reduced by 38% with a central infusion of TGF-β1 (5 μg) for 14 days (Fig. 1B). Paw withdrawal threshold on the ipsilateral side reached 5.57 ± 0.57 g (5 μg TGF-β1) and 5.54 ± 0.44 g (2 μg TGF-β1) vs. 1.51 ± 0.2 g (saline) 13 days following nerve injury (P < 0.001 vs. saline treated) (Fig. 1B). There was no significant difference between 2 μg and 5 μg doses in paw withdrawal threshold and latency. We assume that 2 μg and 5 μg doses have already reached the maximal effect of TGF-β1 on behavioral outcomes.

**Figure 2:**
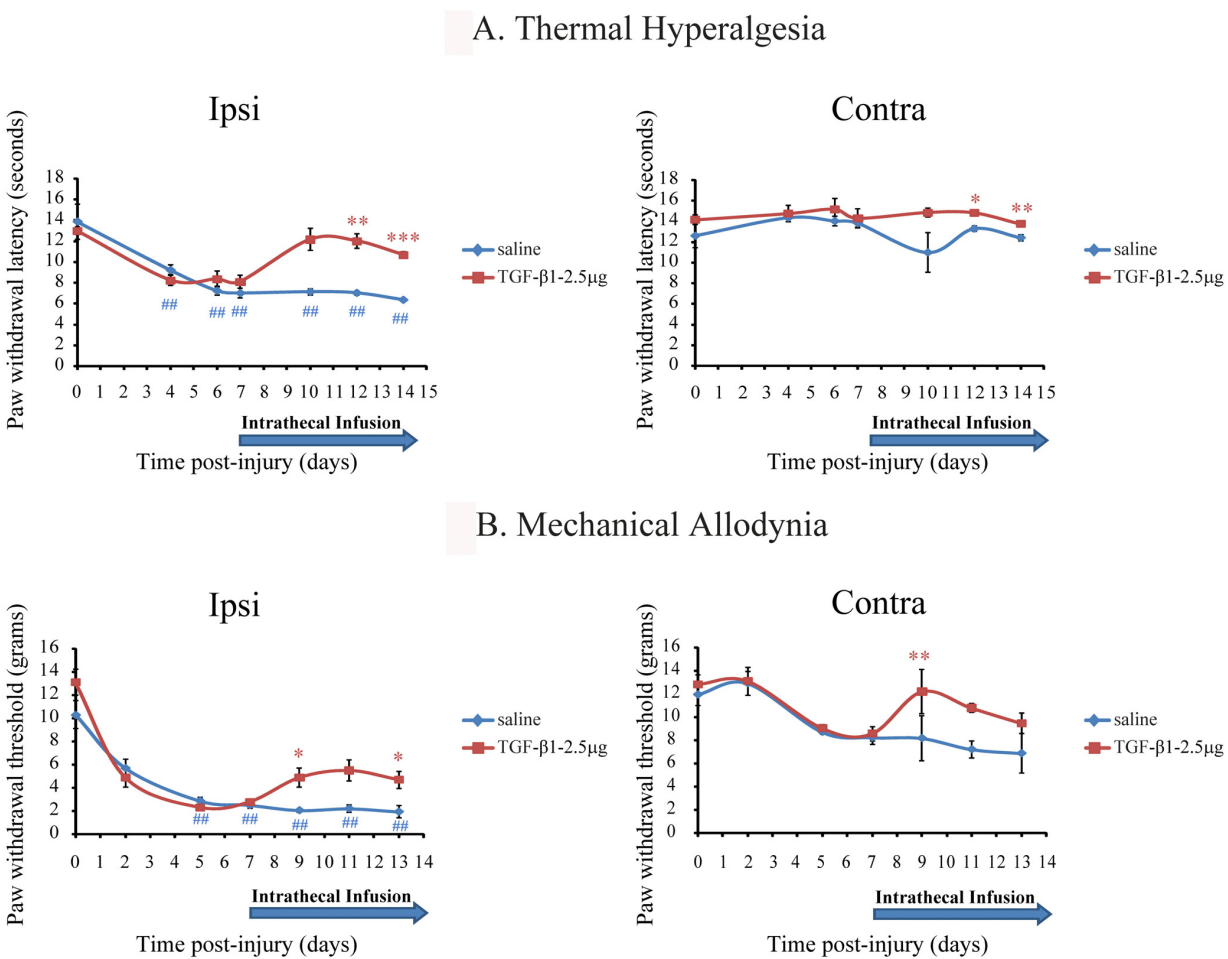
**Delayed administration with TGF-β1 reversed existing nociceptive behaviour**. Rats started to receive TGF-β1 treatment on day 7 post-surgery where both paw withdrawal latency and threshold to heat (**A**) and mechanical stimuli **(B**) respectively, had reached their lowest level. The 7-day infusion of recombinant TGF-β1 (2.5 μg) in the spinal cord of nerve injured-animals (indicated by the blue arrow) resulted in a significant increase of force **(B) **and latency (**A**) in eliciting paw withdrawal compared to saline treated animals. *p < 0.05, **p < 0.01, ***p < 0.001, TGF-β1-treated vs. saline-treated at each time point, ##p < 0.01, each time point vs. presurgery baseline (d0) in the group of injured-saline-treated rats, n = 6 per group.

We also examined the effects of TGF-β1 on already established hypersensitivity following nerve lesion. Implantation of a mini-osmotic pump containing TGF-β1 (2.5 μg) was performed on day 7 post-injury, where both paw withdrawal threshold and latency in response to mechanical and thermal stimuli, respectively, had already reached their lowest level. Intrathecal infusion of TGF-β1 for 7 days produced a 62% reduction in established thermal hyperalgesia (Fig. 2A) and a 28% reversal in mechanical allodynia at ipsilateral sides (Fig. 2B). TGF-β1 also had a slight effect on contralateral side mirror pain (Fig. 2A–B).

### TGF-β1 inhibited nerve injury induced-spinal microglial cell proliferation

**Figure 3:**
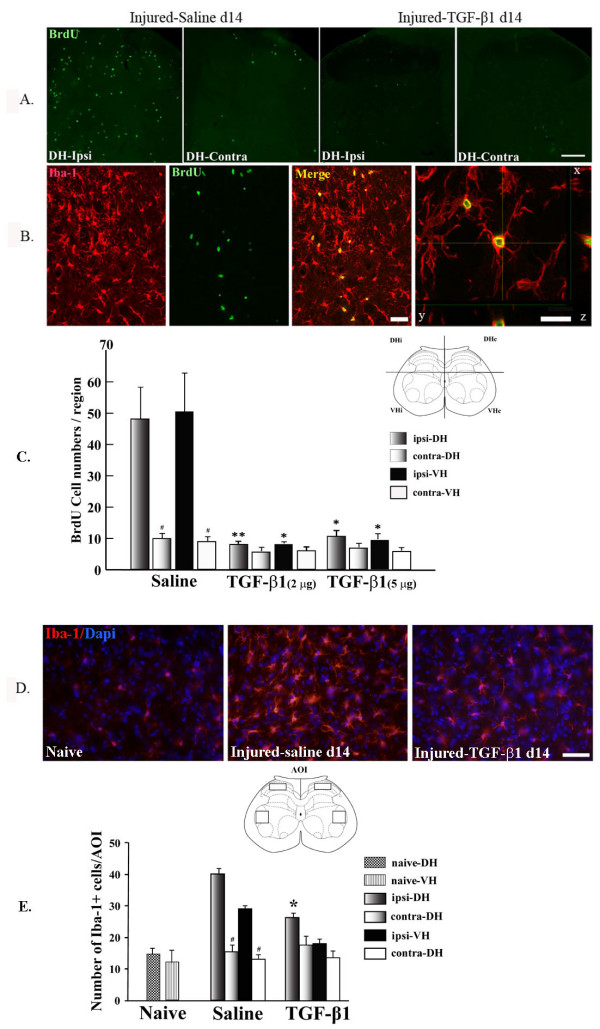
**TGF-β1 inhibited spinal peripheral nerve injury induced spinal microgliosis**. Cell proliferation evoked by peripheral nerve injury was evidenced by an increase in BrdU^+ ^cells in the spinal cord, ipsilateral to the nerve lesion, which was abolished by TGF-β1 treatment **(A)**. Confocal images showing colocalization of BrdU (green nucleus staining) with microglial marker Iba-1 (red cytoplasm stain) revealed that the majority of dividing cells are microglia (**B**). The number of BrdU^+ ^cells in the groups of animals treated or not with TGF-β1 was quantified in four different quadrants (**C**). Representative images of Iba-1^+ ^cells in the spinal cord of naïve rats injured with saline and injured with TGF-β1 treatment depict the effects of TGF-β1 in reducing microglial cell density (**D**); further confirmed by quantitative analysis (**E**). Data are shown as means ± SEM. *p < 0.05, **p < 0.01, TGF-β1 treated vs. saline treated in their respective region. #p < 0.05, ipsi- vs. contra-lateral sides within the same group of injured-saline treated rats. Scale bar: 3A) 100 μm; 3B) 20 and 10 μm; 3D) 20 μm.

We previously reported that peripheral nerve injury (chronic constriction of sciatic nerve with a polyethylene tube) triggers a striking cell proliferation within the spinal cord at the ipsilateral side of the injury. Cell proliferation peaks at day 3 post-injury and persists at high levels at least for 14 days, with most newly generated cells being microglia [7]. In this study, using bromodeoxyuridine (BrdU) as an index of DNA synthesis, we again detected a large number of dividing cells in the lumbar spinal cord, ipsilateral to the sciatic nerve with partial ligation (Fig 3A). The majority of BrdU-incorporated cells were microglia, as they are immunopositive for the microglial marker ionized calcium-binding adaptor molecule 1 (Iba-1) (Fig 3B), indicating that they were newly formed microglia. Intrathecal TGF-β1 treatment successfully inhibited spinal microgliosis following nerve injury, as shown by a significant reduction in the number of BrdU^+ ^cells at the ipsilateral side of the spinal cord (Fig.3A, 3C), and a reduction in density of total Iba-1+ microglial cells (Fig. 3D–E). No significant differences were observed for two different doses (2 μg and 5 μg) of TGF-β1, which suggests the effect of TGF-β1 on the microglial reaction has reached the limit.

### TGF-β1 inhibited spinal microglial and astrocytic activation

**Figure 4:**
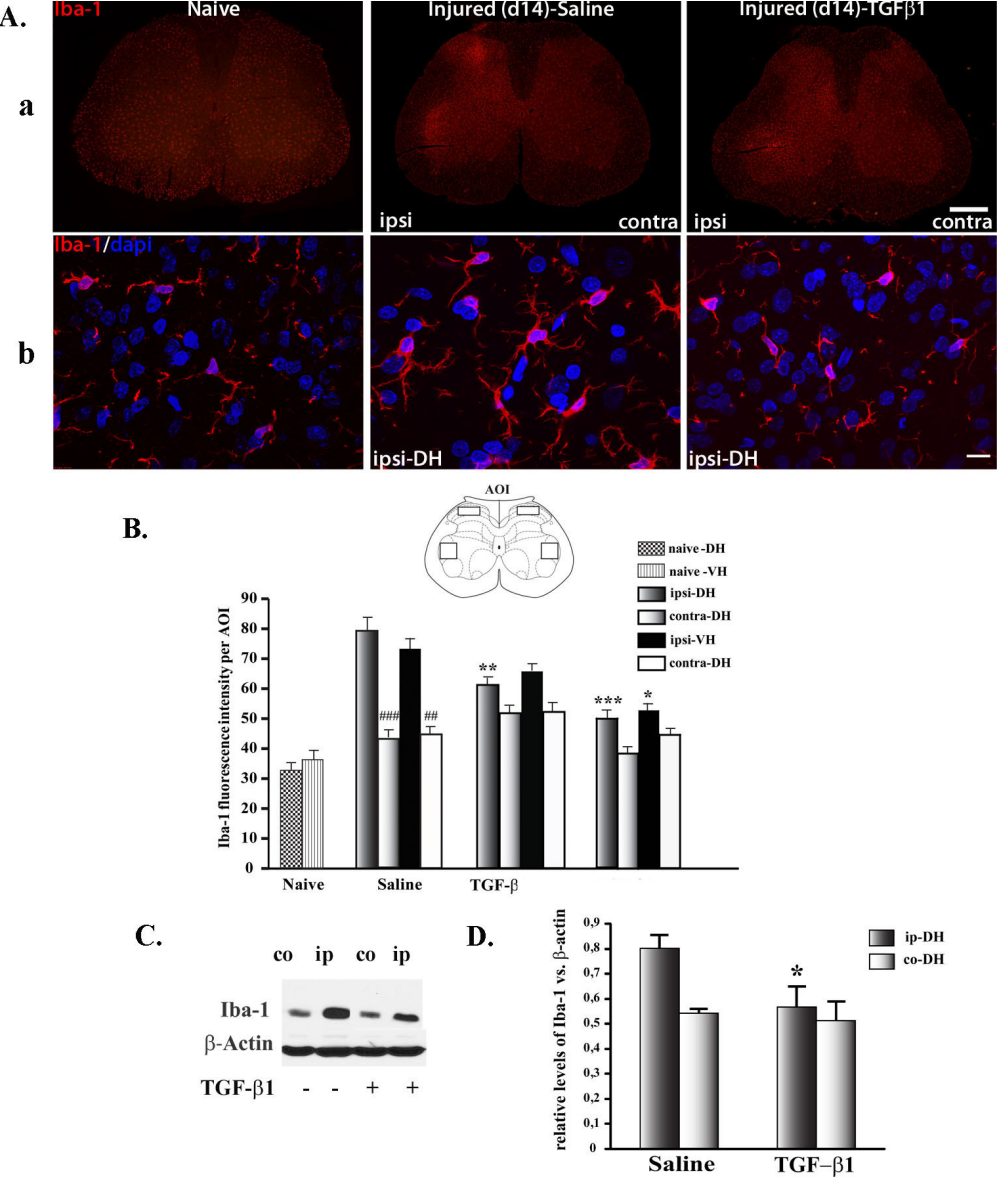
**Role of TGF-β1 in mediating spinal microglial reaction**. **A-a**: Photomicrographs depicting Iba-1 staining in L5 spinal cord section 14 days after nerve injury. To compare with naïve rats, partial sciatic nerve ligation induced a striking increase of immunoreactivity at the ipsilateral side DH and VH. This increase was almost completely abolished in TGF-β1 treated animals. **A-b**: High magnification confocal images demonstrate morphological changes in resting and activated microglia and the effects of TGF-β1 on microglial activation. **B**: Intensity of Iba-1 immunofluorescence determined as average pixel intensity on specific area of interest on L5 section at 14 days after injury. **C-D**: Western Blot analysis demonstrating that up-regulation of Iba-1 protein at the ispilateral side spinal cord was inhibited by TGF-β1 infusion. Tissue samples were collected at 7 days post-injury. Data are shown as means ± SEM. *p < 0.05, **p < 0.01, ***p < 0.001, TGF-β1 treated vs. saline treated in their respective region. ###p < 0.001, ##p < 0.01, ipsi- vs. contra-lateral sides within the same group of injured-saline treated rats. Scale bar: A-a) 1 mm, A-b) 10 μm.

Central TGF-β1 treatment not only inhibited generation of new microglial cells but also prevented activation of existing microglia. In nerve-injured rats receiving saline treatment, there was a strong increase in Iba-1 immunoreactivity (ir) condensed at the ipsilateral side of spinal dorsal horn (DH) and ventral horn (VH) at 14 days post-surgery. This pattern of microglial expression was no longer observed in rats treated with TGF-β1 (Fig. 4A-a). High magnification images revealed that stereotypical changes in microglial morphology during activation were also prevented by TGF-β1. Similar to those in normal animals, Iba-1^+ ^cells in the spinal cord of TGF-β1-treated rats with nerve injury had small cell bodies with long and fine ramifications, whereas microglia in animals without TGF-β1 treatment displayed large cell bodies and enlarged branches following nerve injury (Fig. 4A-b). We quantified the intensity of Iba-1 immunofluorescence 14 days after nerve injury in both groups of rats with 2 μg and 5 μg, and without TGF-β1. Both 2 μg and 5 μg TGF-β1 significantly reduced the effects of nerve injury on Iba-1 ir at the ipsilateral sides DH and VH, with the exception of 2 μg TGF-β1 on the ventral horn, where the effect was not statistically significant (Fig. 4B). We also quantitatively evaluated the effects of TGF-β1 on Iba-1 protein levels at day 7 post-injury with Western Blot. Seven days after nerve injury, the quantity of Iba-1 protein at the ipsilateral side was 1.48 times higher than their contralateral counterpart. Infusion of 1 μg TGF-β1 for one week, similar to the paradigm we used for immunohistochemistry and behavioural studies with 2 μg TGF-β1 infusion for two weeks, significantly reduced the difference between ipsi and contralateral dorsal horns to 1.1 times (Fig. 4C–D).

**Figure 5:**
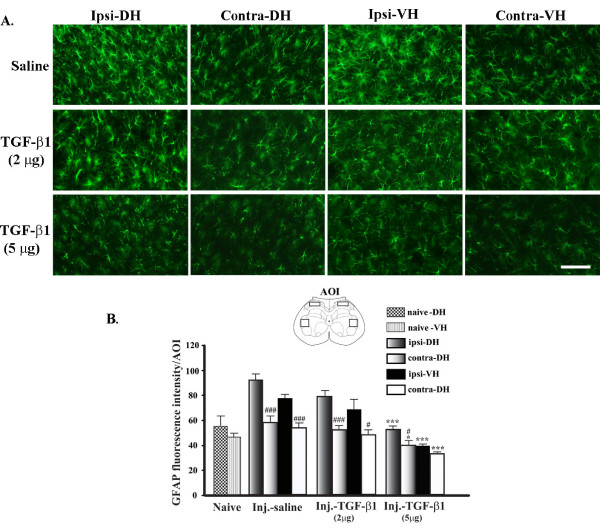
**Role of TGF-β1 in mediating spinal astrocytic reaction**. **A**: Peripheral nerve injury induced an astrocytic activation at the ipsilateral side DH and VH evidenced by an increase of GFAP immunoreactivity. 5 μg TGF-β1 infusion for 14 days significantly reduced the increase in GFAP ir, whereas the effect of 2 μg TGF-β1 was negligible. **B**: Intensity of GFAP signal determined as average pixel intensity at specific area of interests on L5 section at 14 days after injury. Data are shown as means ± SEM. *p < 0.05, ***p < 0.001, TGF-β1 treated vs. saline treated in their respective region. ###p < 0.001, ipsi- vs. contra-lateral sides within the same group. Scale bar: 100 μm.

Next, we examined the effects of TGF-β1 on astrocyte activation in the spinal cord following nerve injury. Spinal astrocytes were labelled with an antibody against glial fibrillary acid protein (GFAP). Changes in GFAP ir and in the morphology of astrocytes within the spinal cord after peripheral nerve injury are depicted in Fig 5A. Partial ligation of sciatic nerve dramatically increased GFAP ir and altered the morphology of astrocytes with enlarged branches at the ipsilateral side of the spinal cord (Fig. 5A, top panels). These effects were attenuated by TGF-β1 in a dose-dependent manner (Fig. 5A–B, middle and lower panels). Treatment with TGF-β1 at 2 μg for 14 days reduced the effect of nerve injury on GFAP ir by 13.9% in DH and 12.3% in VH, whereas at 5 μg TGF-β1 decreased the up-regulation of GFAP ir by 43.1% in DH and 49.12% in VH (Fig. 5A–B), showing a higher dose (5 μg) is more effective in astrocytic response.

### TGF-β1 protected neurons from damage following peripheral nerve injury

**Figure 6:**
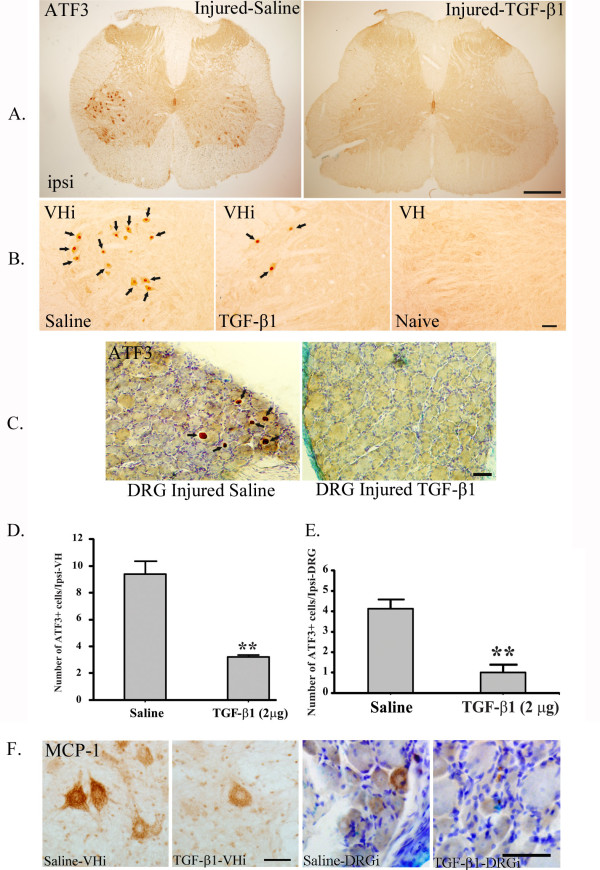
**Neuroprotective effects of TGF-β1**. Partial sciatic nerve ligation caused neuronal damage to both ipsilateral side ventral horn (VHi) motor neurons (**A-B**) and ipsilateral side DRG (DRGi) sensory neurons (**C**), in which ATF3 expression was induced. TGF-β1 treatment prevented neuronal damage by reducing the numbers of ATF3^+ ^cells within the spinal cord and DRG (**A-E**). TGF-β1 treatment also reduced the expression of MCP-1 on VHi and DRGi. Note that both the number of MCP-1^+ ^cells and the intensity of MCP-1 ir were significantly reduced by TGF-β1 (**F**). Data are shown as means ± SEM. *p < 0.05, TGF-β1 treated vs. saline treated in their respective region. Scale bar: A) 200 μm, B-C) 100 μm, F) 50 μm.

Partial sciatic nerve ligation induced the expression of activating transcription factor 3 (ATF3) in the nucleus of sensory neurons in the dorsal root ganglia (DRG) and in the nucleus of motor neurons in the spinal cord (Fig. 6A–C). The number of ATF3^+ ^neurons in the spinal cord was remarkably reduced by 2-week intrathecal TGF-β1 infusion starting on the day of surgery. Fourteen days after sciatic nerve injury, in the ipsilateral side ventral horn, there were 9.36 ± 0.96 ATF3^+ ^cells in the group of rats infused with saline, whereas in rats treated with 2 μg TGF-β1, there were only 3.22 ± 0.15 (2 μg) ATF3^+ ^cells. Fourteen days after sciatic nerve injury, there were 9.36 ± 0.96 ATF3^+ ^cells on the ipsilateral side ventral horn in saline-treated group, whereas in rats treated with 2 μg TGF-β1, there were only 3.22 ± 0.15 ATF3^+ ^cells (Fig. 6D). Although TGF-β1 was introduced intrathecally, expression of ATF3 in the ipsilateral DRG was also affected; 4.2 ± 0.5 ATF3^+ ^cells in saline treated rats vs. 1.00 ± 0.09 ATF3^+ ^cells in TGF-β1 treated rats (Fig. 6E).

As we have previously reported that damaged ATF3^+ ^neurons express chemokine MCP-1 [9] which is a trigger for surrounding microglial activation [8], we further investigated the regulation of MCP-1 expression by TGF-β1. Similar to that of ATF3 regulation, both in the spinal cord ventral horn and in the ipsilateral DRG, the number of MCP-1^+ ^neurons and the intensity of MCP-1 imunoreactivity was significant lower in rats treated with TGF-β1 than those treated with saline (Fig. 6F).

### TGF-β1 reduced spinal inflammatory response following peripheral nerve injury

**Figure 7:**
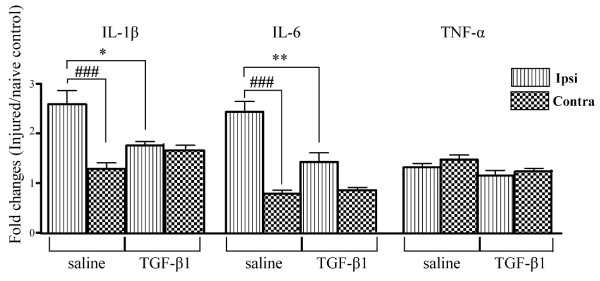
**Anti-inflammatory effects of TGF-β1**. The expression of proinflammatory cytokines IL-1β, TNF-α and IL-6 in rat lumbar spinal cords was measured by real time quantitative PCR. Peripheral nerve injury created an inflammatory reaction within the spinal cord by increasing levels of IL-1β and IL-6 but not TNF-α at the ipsilateral side. TGF-β1 significantly inhibited inflammatory response by reducing the increase of IL-1β and IL-6 expression. Data are shown as means ± SEM. ###p < 0.001, ipsilateral side vs. contralateral side; * p < 0.05, **p < 0.01, TGF-β1 treated vs. saline treated in their respective region.

The role of nerve injury-induced spinal inflammation in the initiation and maintenance of chronic pain has been well documented. To explore whether TGF-β1, a potent anti-inflammatory cytokine, could alter local inflammatory response and thereby explain its potential effect on hypersensitivity, we measured levels of pro-inflammatory cytokines Il-1β, TNF-α and IL-6 transcripts in the spinal cord with quantitative real time-PCR. Relative mRNA levels were normalized with the average of three housekeeping genes (atp5o, Hprt1, G6pdx). Fold changes relative to naïve spinal cord were determined using comparative Ct method [25] and is depicted in Figure 7. Expression levels of IL-1β and IL-6, but not of TNF-α, increased dramatically at the ipsilateral spinal cord following nerve injury. These effects were significantly reduced by TGF-β1 treatment (Fig. 7).

### Expression of TGF-β1, TGF-β1 receptor and the signal transducers Smads in the spinal cord following nerve injury

**Figure 8:**
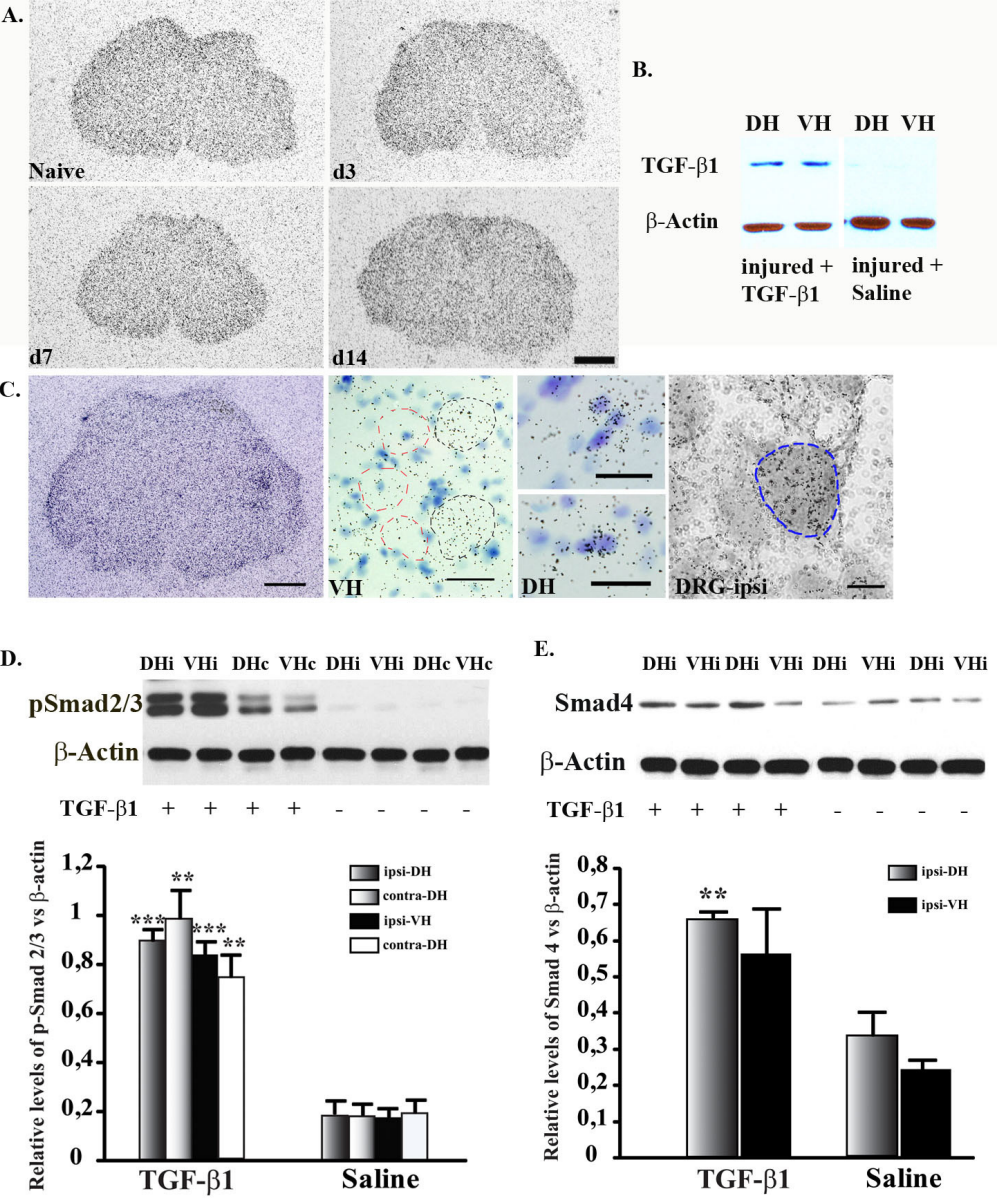
**Expression of TGF-β1, its receptor and signalling proteins-Smads in the spinal cord and DRG**. **A**: The expression of endogenous TGF-β1 mRNA was not detected by *in situ *hybridization at different time points after nerve injury. **B**: Endogenous TGF-β1 protein within the spinal cord was not found by Western Blot, however, intrathecal infusion of recombinant TGF-β1 for 14 days resulted in a stable level in both spinal cord ventral and dorsal horns. **C**: Expression of TGF-β type I receptor (TGF-βRI) mRNA was observed in the spinal cord and DRG. High magnification microscopic analysis revealed that positive signals (silver grains) for TGFβRI mRNA were located on both ventral horn motor neurons and DRG sensory neurons (identified based on the position, appearance and size of cells, encircled in blue), and on some glial cells in both dorsal and ventral horns that were heavily stained with thionine. **D-E**: Recombinant TGF-β1 induced phosphorylation of Smad 2/3 (pSmad2/3) (**D**) and up-regulated the total level of Smad 4 in the spinal cord. Tissue samples were collected 7 days post-injury and TGF-β1 infusion. Data are shown as means ± SEM. **p < 0.01, ***p < 0.001, TGF-β1 treated vs. saline treated in their respective region.

To gain insight into the potential mechanisms of the pleiotropic effects of TGF-β1 in this specific neuropathic condition, we examined the expression of endogenous TGF-β1, the cellular localization of its receptors-TGFβRI/II and the phosphorylation states of receptor-regulated Smad2/3, as well as the expression of the common mediator Smad 4. By using *in situ *hybridization method, TGF-β1 mRNA was not detected within the spinal cord of animals suffering nerve injury from day 0 (naïve) to day 14 (Fig. 8A). Western Blot analysis revealed that endogenous TGF-β1 protein was not detectable in the spinal cord of rats following nerve injury. However, chronic intrathecal infusion of recombinant TGF-β1 resulted in a stable and similar level of the protein at both dorsal and ventral horns (Fig. 8B). This expression pattern confirms that the pleiotropic cellular effects of TGF-β1 and the subsequent impact on behavioural outcomes are essentially mediated through exogenous TGF-β1, excluding the interference of endogenous TGF-β1.

TGFβRI mRNA was up-regulated in the spinal cord and DRG, ipsilateral to the nerve injury (Fig. 8C). Positive signals (silver grains) for TGFβRI mRNA were observed on the ventral horn motor neurons and DRG sensory neurons (identified based on the position, appearance and size of cells) and on some glial cells in both dorsal and ventral horns, which were heavily stained with thionine (Fig. 8C). TGFβRII mRNA was not detected by *in situ *hybridization within the spinal cord parenchyma following nerve injury but was found in the plexus and ventricle in the normal brain (data not shown).

The binding of ligand TGF-β1 to a heteromeric receptor complex consisting of TGFβRI and TGFβRII, leads to activation of the Smads signalling pathway, including receptor-regulated Smads (R-Smads)-Smad2/3 and the common mediator (Co-Smad)-Smad 4. This intracellular cascade is critical to the pleiotropic action of TGF-β1 superfamily responses. To determine whether Smad signalling was activated in the spinal cord of rats with nerve injury following TGF-β1 challenge, we measured protein levels of phosphorylated Smad 2/3 and Smad 4 in the spinal cord with Western Blot. Both p-Smad 2/3 (Fig. 8D) and Smad 4 (Fig. 8E) were up-regulated at the ipsilateral spinal cord after TGF-β1 treatment.

## Discussion

Peripheral nerve injury produced a long lasting mechanical allodynia and thermal hyperalgesia. This hypersensitive state was accompanied by early and robust spinal microglial reaction, including a striking spinal microgliosis, persistent spinal astrocyte activation and a spinal inflammatory reaction, evidenced by an increase in proinflammatory cytokine levels. We investigated the effects of anti-inflammatory cytokine TGF-β1 delivered intrathecally on neuropathic pain behaviour. Our findings demonstrate that TGF-β1 is effective, not only in preventing, but also in reversing the hypersensitivity evoked by damage to the peripheral nerve. Three salient features of TGF-β1 were observed in this study: 1) TGF-β1 exerts potent neuroprotective effects that minimize neuronal damage following peripheral nerve injury; 2) TGF-β1 inhibits spinal microglial and astrocytic activation; and 3) TGF-β1 decreases nerve lesion induced up-regulation of pro-inflammatory cytokine IL-1β and IL-6 within the spinal cord.

Although it has been reported that TGF-β1 could trigger neuronal cell death [26,27], numerous *in vitro *and *in vivo *studies have shown a protective effect of TGF-β1 against various toxins and injurious agents [18,28]. TGF-β1 prevented neuronal damage when delivered intracerebrally or using viral vectors into rodent brains after ischemic insults [28]. TGF-β1 may have similar effects in neurodegenerative diseases such as Alzheimer's disease and Parkinson's disease [29-31]. The roles of TGF-β1 in maintaining neuronal integrity and survival were also evidenced with molecular approaches. TGF-β1^-/- ^mice showed increased numbers of apoptotic neurons, reduction in neocortical presynaptic integrity, and died at the age of 3–4 weeks [32]. Heterozygous knock out (TGF-β1^-/+^) mice showed increased susceptibility to excitotoxic injury and neurodegeneration, whereas transgenic over-expression of TGF-β1 prevented degeneration after excitotoxic injury [32]. ATF3 is a member of the activating transcription factor/cAMP-responsive element binding protein family (ATF/CREB family) and is recognized as a useful marker for nerve injury. It is detected in DRG sensory neurons and spinal cord motor neurons within hours of axotomy [33] and lasts for at least 80 days post-injury [34]. In this study, recombinant TGF-β1 delivered into the spinal cord significantly reduced the number of ATF3^+ ^cells. This is consistent with previous findings showing neuroprotective effects of this cytokine, which could be a direct pathway in restoring neuronal damage resulting from nerve injury. TGF-β1 delivered into the spinal cord not only protected spinal motor neurons but also sensory neurons in DRG following a partial ligation on the sciatic nerve. This finding further confirms that intrathecal injection is an effective delivery method for drugs that interfere with the expression and function of target genes in both peripheral sensory ganglia and their postsynaptic targets in the spinal cord [35]. Different mechanisms have been postulated to explain how TGF-β1 protects neurons against injury and degeneration. A particularly striking example is the interaction of TGF-β1 with some other neurotrophins. TGF-β1 may synergize with these neurotrophins by dramatically increasing their potency or they may be required to mediate at least some of the effects of NGF, BDNF and NT-3, as well as FGF-2 and GDNF [20]. However, in this specific case where exogenous TGF-β1 significantly improved the nerve injury induced neuropathic pain, whether and how TGF-β1 interacted with other trophic factors is not clear and further studies are needed for clarification. The detection of TGFβRI mRNA on neurons indicates that TGFβ-1 alleviates neuropathic pain at least partially through direct action on damaged neurons.

TGF-β1 could also act directly on the CNS glial cells. Several lines of evidence support the notion of TGFβ-1 is a suppressor of functions of activated microglia. TGF-β1 inhibits proliferation of microglia and blocks free radical induction [21,36]. Moreover, TGF-β1 selectively induces apoptosis of microglia through a Bcl-2 independent mechanism [36]. In most situations, TGF-β1 inhibits growth of astrocytes and affects their morphology and mobility [37,38]. Recent studies in knock-out mice demonstrated that deficiency in TGF-β1 caused gliotic changes throughout the CNS, affecting astrocytes, microglia and perivascular macrophages [39]. These changes include increases in GFAP-ir, two to three-fold expansion in the number of perivascular macrophages with a strong immunoreactivity for MHCII, and a proliferation of microglia that express a panel of phagocytic inflammatory markers [39]. Transection of the facial nerve in TGF-β1^-/- ^provoked a more pronounced astrogliosis and a dramatic increase in phagocytosis-related microglial markers including Iba-1 [39]. Results from our investigations clearly demonstrate that injury to the sciatic nerve induced a persistent hypersensitivity of the paw accompanied by spinal microglial and astrocytic activation and that TGF-β1 treatment attenuated or abolished the microgliosis and activation of microglia and astrocytes following nerve injury. Inhibitory effects of TGF-β1 on microglia and astrocytes could occur by directly targeting spinal glial cells where the TGFβ type I receptor was detected. This could also be possible through indirect pathway via protection of neuronal damage. Our previous results revealed that, following sciatic nerve injury, damaged ATF3^+ ^DRG sensory neurons and spinal cord motor neurons were induced to express chemokine MCP-1 [9]. Neuronal MCP-1 is critical for both resident spinal microglial activation and peripheral macrophage infiltration and in the development of neuropathic pain [8]. Our current results further confirmed that reducing the numbers of ATF3+ neurons by TGF-β1 also reduced the production of MCP-1. These findings suggest that in addition to direct action on glial cells, TGF-β1 could attenuate neuropathic pain by suppressing neuronal MCP-1 expression, consequently inhibiting neuron-glia signalling.

CNS glial cells are considered immune-competent cells. Immediately following injury directly to the CNS or remotely to the PNS, damage-sensing glial cells become locally activated and release inflammatory mediators to defend against potential invading pathogens. Several proinflammatory cytokines and chemokines have been implicated in altered nociceptive processing via direct action on primary afferent neurons or through indirect activation of signalling pathways in immune/glial cells, resulting in an excitatory positive feedback loop in the pain pathway. It has been recently demonstrated that TNF-α and IL-6 modulate excitatory and inhibitory synaptic transmission respectively, whereas IL-1β controls both excitatory and inhibitory synaptic transmission [40]. Spinal IL-1β was also shown to induce the transcription of pronociceptive genes such as COX-2 in the spinal cord [41]. All three proinflammatory cytokines induced phosphorylation of the transcription factor CREB, which is essential to the maintenance of long-term neural plasticity in dorsal horn neurons [42]. By using real time PCR, we observed a significant increase in IL-1β and IL-6 gene expression at the ipsilateral side of the spinal cord. Although the increase of TNF-α transcript within the spinal cord was not detected, one cannot exclude the possibility that up-regulation of TNF-α gene expression occurred in the DRG and the protein was then transported into the central terminals. We speculate that local spinal inflammation enhances central sensitization and is critical to the development of neuropathic pain. Our data strongly suggest that exogenous TGF-β1 mitigates neuropathic pain through inhibition of the production of pro-inflammatory cytokines.

TGF-β1 uses a well-characterized signal transduction pathway that extends from the cell membrane to the nucleus. Active TGF-β1 ligand binds to TGFβRII to form a complex with TGFβRI. Activation of a receptor complex leads to phosphorylation of Smads proteins. Thus, expression of both receptors is required for TGFβ-1 signalling. However, in *in vivo *conditions, parallel regulation of the ligand and their receptors and co-expression of two receptor subtypes on the same cell are not always seen. By using *in situ *hybridization, we detected expression of TGFβRI in both neurons and glial cells at the ipsilateral side lumber spinal cord without the presence of TGFβRII. One possible interpretation is that TGFβRII, which is often constitutively active [17], could be present at levels undetectable by the method used in this study; however, it remained functional. This idea is supported by our results on Smad2/3 and Smad4. Peripheral nerve damage altered spinal cord homeostasis, affecting both neurons and glia; and exogenous TGF-β1 activated the signalling pathway (TGFβRI/TGFβRII receptor complex and Smad) and therefore contributed to alleviating chronic pain through its pleiotropic effects on different types of cells.

Our study is the first demonstration of the significant impact of TGF-β1 in normalizing behavioural pain and other changes associated with neuropathic pain states. Pleiotropic effects of TGF-β1 are sufficient to prevent onset of abnormal pain states arising from the neuropathy. In addition, a delayed treatment paradigm in which TGF-β1 administration began after the establishment of neuronal damage, glial activation and local inflammatory reaction, demonstrated that TGF-β1 can indeed reverse neuropathic pain. In clinical settings, drug treatment is initiated after onset of neuropathic pain. Therefore, the ability of an agent to reverse established pain may translate into clinical usefulness. Although it is well known that TGF-β1 is a pleiotropic protein, our results clearly demonstrate that its pleiotropic role can have a positive impact in spinal cord homeostasis and on hypersensitivity following nerve injury, where the pathology is a result of dysfunction at different cellular levels. Functional improvements may be attributed to the ability of TGF-β1, acting as a multipotent cytokine, to support neuronal survival, modulate inflammatory response and orchestrate neuronal and glial response to injury. The efficacy of exogenous TGF-β1 in blocking nerve injury-induced pain elicits TGF-β1 as a potential therapeutic agent for neuropathic pain. Consequently, due to the pleiotropic effects of TGF-β1, a detailed investigation of the off-target side effects is necessary to validate TGF-β1 as a therapeutic agent.

## Methods

### Animals

Adult male Sprague-Dawley rats (Charles River, Quebec, Canada) were used and weighed 250–275 g at time of surgery. Before surgery, they were acclimatized to standard laboratory conditions (14-h light, 10-h dark cycle) and given free access to rat chow and water. All protocols were performed in accordance with guidelines from the Canadian Council on Animal Care and were approved by the McGill University Animal care Committee.

### Nerve injury and behavioural studies

#### Peripheral nerve injury

Rats were anaesthetized with a mixture of ketamine and xylazine (100 mg/kg body weight, i.p.). The left common sciatic nerve was exposed via blunt dissection through the biceps femoris muscle. The nerve was isolated from surrounding connective tissue and approximately 4–6 mm of the nerve was elevated minimally and partial sciatic nerve ligation was conducted according to the method described by Seltzer et al. [24]. Briefly, the dorsum of the nerve was carefully freed from surrounding connective tissues at a site near the trochanter. A 6-0 suture was inserted into the nerve with a 3/8 curved, reversed-cutting mini-needle (Tyco Health Care, Mississauga, Ontario, Canada) and tightly ligated so that the dorsal one-third to one-half of the nerve thickness was trapped in the ligature. The muscle and skin layers were closed under aseptic conditions with two muscle sutures (4-0) and three to four skin sutures (4-0). Sham-operated rats underwent the same surgical procedure but the nerve was exposed and left intact. Survival times were 7 and 14 days post-surgery. A group of naive rats was included in the protocol to obtain basal levels of certain gene and protein expression.

#### Behavioural analysis

Animals were habituated to the testing environment daily for at least two days before baseline testing. All animals were assessed for mechanical allodynia and thermal hyperalgesia of both hind paws before surgery and from days 2–3 after injury until they were killed for histological studies. The investigator was totally blinded to the treatments the rats received. Mechanical sensitivity was assessed using calibrated von Frey Hairs as described by Chaplan et al [43]. Animals were placed in boxes on an elevated metal mesh floor and allowed 40 to 60 min for habituation before testing. A series of von Frey filaments with logarithmically incrementing stiffness (Stoelting) was applied perpendicular to the mid-plantar region of the hind paw. The 50% paw withdrawal threshold was determined using Dixon's up-down method as previously described [44]. Thermal hyperalgesia was measured using paw withdrawal test. Animals were placed on a glass floor within Plexiglass cubicles. After habituation, a focused high-intensity projector lamp was directed below onto the mid-plantar surface of the hind paw and reaction time (withdrawal latency of the hind paw) of the rat was recorded automatically [45]. The commercial device (IITC Model 336) was calibrated so that the pre-surgical baseline paw withdrawal latencies were approximately 10–12 seconds. Twenty seconds was used as a cut-off time to avoid damage to the animal's skin. The measurements were repeated four times, at 3 min intervals on each paw, and the initial pair of measurements was not used. The average of the three remaining pairs of measurements was taken as data. Efficacy of TGF-β1 was determined according to the following formula: Mean_TGF-β1_-Mean_control(saline)_/Mean_naive(baseline)_-Mean_control(saline) _× 100%.

### TGF-β1 administration

Rat recombinant TGF-β1 (Peprotech, RockyHill, New Jersey, U.S.A.) was delivered into nerve injured and sham-operated rats with an intrathecal catheter driven by a mini-osmotic pump (Alzet, Palo Alta, CA). The catheter and pump were implanted according to the procedure originally described for chronic catheterization of the rat spinal cord [46]. Animals were anaesthetized with a mixture of ketamine/xylazine (100 mg/kg, intraperitoneally). A midline incision was made to expose the atlanto-occipital membrane. The membrane was pierced and the catheter was passed intrathecally to a distance of 8.5 cm, i.e., caudal to the level of the atlanto-occipital junction. The coiled part of the system with the pump attached was then implanted subcutaneously in a pouch to lie behind the shoulders. Rats showing any signs of motor impairment were excluded from the protocol. Alzet osmotic mini-pumps were filled with 0.9% saline or TGF-β1 and primed in a 37°C bath over night. All intrathecal tubings and pumps were verified at the end of experiments during tissue collection. Animals having a defective intrathecal catheterization (either disconnected or clogged) were eliminated from the study.

#### TGF-β1 treatment paradigms

##### Experiment 1

Intrathecal catheters and osmotic pumps were implanted at the time of nerve ligation surgery to determine the impact of TGF-β1 on the development of neuropathic pain. Rats received saline or TGF-β1 treatment (2 μg and 5 μg, respectively with Alzet pumps 2002D) for 14 days (day 0–14) (n = 6 per group). In our pilot study with 0.5–1 μg TGF-β1 infusion, we observed partial effects on behavioural and microglia response (data not shown). To examine whether this is the maximal effect, we increased the dose of TGF-β1 to 2 μg and 5 μg. Animals were perfused at day 14 post-injury and treatment for histological analysis. In another group, rats received saline or TGF-β1 infusion (1 μg with Alzet pumps 1007D) for 7 days (day 0–7) (n = 6 for saline and n = 12 for TGF-β1 infusion). Tissues collected from this group of rats were subjected to real-time quantitative PCR and Western Blot analysis. Naïve and Sham operated rats (n = 3 per group) were also included in the protocol.

##### Experiment 2

To determine whether TGF-β1 could also reverse already established neuropathic hypersensitivity, TGF-β1 (2.5 μg using Alzet pumps 1007D) was delivered intrathecally starting from day 7 post-injury, when both mechanical allodynia and hyperalgesia had reached their lowest level. Treatment lasted for 7 days (day 7–14) (n = 3 for saline and n = 4 for TGF-β1 infusion).

### Tissue preparation

#### For histological studies

Rats were deeply anaesthetized with ketamine/xylazine and then perfused transcardially with 0.9% saline followed by 4% paraformaldehyde (PFA) in 0.1 M sodium phosphate buffer (pH 7.4). Lumbar spinal cords and L4–L6 dorsal root ganglia (DRG) were removed and placed in the same fixative overnight, then transferred to 30% sucrose for cryoprotection. Frozen spinal cords and DRG were cut transversely into 30-μm-thick sections on a sliding microtome, collected in an anti-freeze solution [0.05 M sodium phosphate buffer (pH 7.3) containing 30% ethylene glycol and 20% glycerol] and stored at -20°C until use.

#### For real time PCR experiments

Rats were deeply anaesthetized with isoflurane and decapitulated, then lumbar spinal cords were quickly removed and dissected into ipsilateral and contralateral portions and then snap frozen in liquid nitrogen and stored at -80°C until use.

#### For Western Blot studies

Animals were transcardially perfused with Ringer Solution and lumbar spinal cords were removed and dissected into quadrants of 1) ipsilateral dorsal horn (DHi), 2) contralateral dorsal horn (DHc), 3) ipsilateral ventral horn (VHi), and 4) contralateral ventral horn (VHc), and then frozen in liquid nitrogen stored at -80°C until use.

### Cell proliferation study

The thymidine analog bromodeoxyuridine (BrdU) was used to label proliferating cells in the spinal cord after sciatic nerve injury. BrdU was injected intraperitoneally (100 mg/Kg body weight) at day 3 post-injury; the time when peripheral nerve injury-induced cell proliferation within the spinal cord reached its peak [7]. Animals were perfused transcardially with 4% paraformaldehyde (PFA) for tissue collection. To allow for detection of cells incorporated with BrdU, free-floating sections were pre-treated with 50% formamide in 2 × SSC for 2 h at 65°C, followed by 15 min in 2 × SSC at room temperature, and 30 min in 2N HCl at 37°C for 10 min in 0.1 M borate buffer at room temperature. A polyclonal goat anti-rat antibody against BrdU (1:250; Accurate Chemicals, Westbury, NY) was incubated with tissue sections for 48 h at 4°C. After primary antibody incubation, sections were incubated in Alexa 488-conjugated goat anti-rat IgG (1:250; Invitrogen, Carlsbad, CA) for 1 h. Sections were mounted onto slides and coverslipped with Vectashield mounting medium (Vector Laboratories, Burlingame, CA).

### Immunohistochemistry

Regular immunofluorescent staining was performed to characterize the spinal glial cell reaction to peripheral nerve injury and to TGF-β1 chronic infusion. Free-floating sections were incubated overnight at 4°C with the following antibodies: rabbit anti-ionized calcium-binding adaptor molecule 1 (Iba-1) polyclonal antibody (for microglia and macrophages, 1:1000; Wako Chemicals, Richmond, VA); and rabbit anti-glial fibrillary acid protein (GFAP) polyclonal antibody (for astrocytes, 1:1000; DakoCytomation, Carpinteria, CA); followed by a 60-min incubation at room temperature in fluorochrome-conjugated goat secondary antibody. 4',6-Diamidino-2-phenylindole dihydrochloride (Dapi) was also used as a nuclear counterstain (1:10000, Sigma).

Some spinal cord and DRG sections were processed by avidin-biotin method using peroxidase as a substrate to detect damaged neurons labelled by antibodies against activating transcription factor 3 (ATF3) and monocyte chemoattractant protein-1 (MCP-1). Briefly, sections were rinsed with TBS and incubated with goat anti-rabbit ATF3 antibody (1:500, Santa Cruz biotechnology, USA,) or goat anti-rabbit MCP-1 antibody (1:2000, Peprotech, RockyHill, New Jersey, U.S.A) over night at 4°C and incubated sequentially with a biotinylated secondary antibody (Vector Laboratories, Canada), followed by an avidin-biotin-peroxidase complex (Vectostain ABC Elite Kit, Vector Laboratories). After several washes in TBS, tissue sections were reacted in 0.05% diaminobenzidine and 0.003% hydrogen peroxide and some sections were counterstained with thionin.

### *In situ *hybridization

Detection of mRNAs encoding TGF-β1, TGFβRI and TGFβRII was performed on lumbar spinal cord and DRG sections using 35S-labeled riboprobes. Riboprobe synthesis and hybridization were performed as per a previously described protocol [47]. Briefly, plasmids were linearized and sense and anti-sense cRNA probes were synthesized with appropriate RNA polymerase as described in Table 1. Sections were postfixed in 4% PFA and digested by proteinase K (10 μg/ml, at 37°C for 20 min), after which spinal cord sections were rinsed in water and by a solution of 0.1 M triethanolamine (TEA, pH 8.0), acetylated in 0.25% acetic anhydride in 0.1 M TEA and then dehydrated. Hybridization of the sections by riboprobe involved a 10^6 ^cpm/ml/slide of hybridization mixture and incubation at 55°C overnight in a slide warmer. Slides were rinsed in standard saline citrate (1 × SSC: 0.15 M NaCl, 15 mM trisodium citrate buffer, pH 7.0) and digested by RNase A at 37°C (20 μg/ml), rinsed in descending concentrations of SSC, and dehydrated through graded concentrations of ethanol. Sections were exposed to x-ray film (BioMax, Kodak, Rochester, NY) for 2–3 days and dipped in NTB2 nuclear emulsion (diluted 1:1 with distilled water, Kodak). Slides were kept at 4°C for 3–5 weeks safe from light and developed in D19 developer (Kodak).

**Table 1:** Plasmids and enzymes used for synthesis of the cDNA probes

**Plasmid**	**Vector**	**Insert**	**Antisense probe**	**Sense probe**	**GenBank Reference**
Topo+TGFβRI	BluntIItopo	597 pb	Pst1/Sp6	HindIII/T7	NM-009370
Topo+TGFβRII	BluntIItopo	592 pb	BamHI/T7	XbaI/Sp6	D32072
pBSKS+ TGF-β1	pBSKS+	1,173 pb	EcorI/T3	XhoI/T7	BC013738

### Western Blot Analysis

Tissues were homogenized in a lysis buffer containing a cocktail of proteinase and phosphatase inhibitors (Roche, Indianapolis, USA). Protein concentrations were determined by BCA Protein Assay (Pierce) and 15 μg of proteins were loaded and separated on SDS-PAGE gel (12%). After the transfer, blots were incubated overnight at 4°C with the following antibodies as per manufacturer's instructions: goat anti-p-Smad2/3 (1:500, Santa Cruz); goat anti-Smad4 (1:250, Santa Cruz); rabbit anti-Iba-1 (1:500, Wako); and goat anti-TGF-β1 (1:500, Santa Cruz). For loading control, blots were probed with β-actin antibody (1:10000, Sigma). Density of specific bands from Western Blotting was quantified with a computer-assisted imaging analysis system (Image Pro Plus).

### RNA extraction and real-time quantitative PCR

Total RNA was extracted from spinal cords using Qiazol (Invitrogen) followed by digestion with RQ1 RNase-Free DNase RNeasy kit (Qiagen) to remove DNA contamination. Synthesis of cDNA from total RNA was performed with SuperScript II Reverse Transcriptase (Invitrogen). Primers (see Table 2) were chosen using the GeneTools software (Biotools Inc.) and their specificity was verified using the GenBank database from the NCBI website. Nineteen samples were analysed (5 TGF-β1-treated animals; 3 saline-treated animals; 3 naive animals). Experiments were performed in duplicate using the LightCycler 480 Real-Time PCR Detection System (Roche Diagnostics). Levels of target mRNAs were normalized to the average of the three housekeeping genes; Atp50, Hpt1 and G6pdx. Fold changes versus naive animals in their respective ipsi and contralateral sides were analyzed using the comparative Ct (dCT) method [25].

**Table 2:** Detailed information on the selection of primers for real-time RT-PCR experiments

**Gene**	**Description**	**GenBank**	**Region**	**Size (pb)**	**Primers sequence: 5' → 3' (S/AS)**
IL-1β	Rattus norvegicus interleukin 1 beta	NM_031512	456–639	184	CTCGTGCTGTCTGACCCATGT/TGGGTGTGCCGTCTTTCATCA

TNF-α	Rattus norvegicus tumor necrosis factor	NM_012675	426–668	243	CGTCGTAGCAAACCACCAAGC/ATGGCGGAGAGGAGGCTGACT

IL-6	Rattus norvegicus interleukin 6	NM_012589	474–735	262	ACAAAGCCAGAGTCATTCAGAGCAA/AATGTCCACAAACTGATATGCTTAGGC

Atp5o	Rattus norvegicus ATP synthase, H+ transporting, mitochondrial F1 complex, O subunit	NM_138883	223–453	231	GGTGTCCCTTGCTGTTCTGAA/TCTAAAGGAAACGCTGTGGTCA

Hprt1	Rattus norvegicus hypoxanthine guanine phosphoribosyl transferase 1	NM_012583	38–266	229	CAGTCCCAGCGTCGTGATTAGT/ATCCAGCAGGTCAGCAAAGAACT

G6pdx	Rattus norvegicus glucose-6-phosphate dehydrogenase X-linked	NM_017006	1429–1664	236	GTATCTTCACACCATTGCTGCA/TTAGATGGTGAGAAGGGCAGAT

### Image processing and analysis

Images were acquired using either an Olympus BX51 (Tokyo, Japan) microscope equipped with a colour digital camera (Olympus DP71) or an Olympus confocal laser-scanning biological microscope (Fluoview 1000). Colocalization was ensured with confocal Z stacks at 0.8 μm intervals and visualization in three-dimensional orthogonal planes. Quantitative analysis of the immunofluorescence intensity was performed on images digitized using a constant set of parameters (exposure time, gain, and post-image processing) with special attention to avoid signal saturation. We measured the intensity of Iba-1 and GFAP immunofluorescence as the average fluorescence intensity within four areas of interest (AOI) defined as: 2 rectangles (100 × 300 μm/each) on the dorsal horn (DH) (lamina I–IV) and 2 rectangles (261 × 370 μm/each) on the ventral horn (VH) (lamina IX), on both sides relative to the side of injury. Fluorescence intensity was measured using Image Pro Plus 6.2 for Windows (Media Cybernetics Inc.). The total number of BrdU^+ ^cells and Iba-1^+ ^microglial cells was counted by blinded investigators in four different regions: ipsilateral DH (DHi), contralateral DH (DHc), VHi, and VHc. Only uniformly BrdU^+^-labelled nuclei and Iba-1^+ ^cells showing a positive nucleus stained with Dapi were considered for quantification. Samples from 4–6 sections per rat and 6 rats per group were included for each quantitative analysis.

### Statistical analysis

All data are presented as means ± SEM. Statistic significance was determined using: 1) for behavioural analysis in Fig 1 and Fig 2, one way ANOVA followed by Dunnet's test for the changes of all time points vs. pre-surgery baseline; unpaired t-test for the difference between groups (TGF-β1-treated vs. saline-treated at each time point); 2) paired t-test for the difference between ipsi- and contra-lateral sides within the same group; and 3) unpaired t-test for the difference between groups (TGF-β1-treated vs. saline-treated in their respectively DH and VH region. The criterion for statistical significance was p < 0.05.

## Competing interests

The authors declare that they have no competing interests.

## Authors' contributions

SE carried out surgery, behavioral testing, histological studies and Western Blot. XQS participated in behavioral testing and histological studies. AH participated in histological studies. HL was responsible for the experiments related to molecular biology. SE also participated in data analysis and preparation of the manuscript. ZWZ contributed to the conception of the study and preparation of the manuscript. JZ conceived the project, lead the experimental design and data analysis and wrote the manuscript. All authors read and approved the final manuscript.

## References

[B1] Sindrup SH, Jensen TS (1999). Efficacy of pharmacological treatments of neuropathic pain: an update and effect related to mechanism of drug action. Pain.

[B2] Watson CP (2000). The treatment of neuropathic pain: antidepressants and opioids. Clin J Pain.

[B3] Scholz J, Woolf CJ (2007). The neuropathic pain triad: neurons, immune cells and glia. Nat Neurosci.

[B4] Colburn RW, Rickman AJ, DeLeo JA (1999). The effect of site and type of nerve injury on spinal glial activation and neuropathic pain behavior. Exp Neurol.

[B5] Zhang J, Hoffert C, Vu HK, Groblewski T, Ahmad S, O'Donnell D (2003). Induction of CB2 receptor expression in the rat spinal cord of neuropathic but not inflammatory chronic pain models. Eur J Neurosci.

[B6] Fu KY, Light AR, Matsushima GK, Maixner W (1999). Microglial reactions after subcutaneous formalin injection into the rat hind paw. Brain Res.

[B7] Echeverry S, Shi XQ, Zhang J (2008). Characterization of cell proliferation in rat spinal cord following peripheral nerve injury and the relationship with neuropathic pain. Pain.

[B8] Zhang J, Shi XQ, Echeverry S, Mogil JS, De Koninck Y, Rivest S (2007). Expression of CCR2 in both resident and bone marrow-derived microglia plays a critical role in neuropathic pain. J Neurosci.

[B9] Zhang J, De Koninck Y (2006). Spatial and temporal relationship between monocyte chemoattractant protein-1 expression and spinal glial activation following peripheral nerve injury. J Neurochem.

[B10] Nesic O, Lee J, Johnson KM, Ye Z, Xu GY, Unabia GC, Wood TG, McAdoo DJ, Westlund KN, Hulsebosch CE, Regino Perez-Polo J (2005). Transcriptional profiling of spinal cord injury-induced central neuropathic pain. J Neurochem.

[B11] Mantyh PW, Clohisy DR, Koltzenburg M, Hunt SP (2002). Molecular mechanisms of cancer pain. Nat Rev Cancer.

[B12] Scholz J, Woolf CJ (2007). The neuropathic pain triad: neurons, immune cells and glia. Nat Neurosci.

[B13] Milligan ED, Watkins LR (2009). Pathological and protective roles of glia in chronic pain. Nat Rev Neurosci.

[B14] Suter MR, Wen YR, Decosterd I, Ji RR (2007). Do glial cells control pain?. Neuron Glia Biol.

[B15] Blobe GC, Schiemann WP, Lodish HF (2000). Role of transforming growth factor beta in human disease. N Engl J Med.

[B16] Bottner M, Krieglstein K, Unsicker K (2000). The transforming growth factor-betas: structure, signaling, and roles in nervous system development and functions. J Neurochem.

[B17] Dennler S, Goumans MJ, ten DP (2002). Transforming growth factor beta signal transduction. J Leukoc Biol.

[B18] Flanders KC, Ren RF, Lippa CF (1998). Transforming growth factor-betas in neurodegenerative disease. Prog Neurobiol.

[B19] Lippa CF, Smith TW, Flanders KC (1995). Transforming growth factor-beta: neuronal and glial expression in CNS degenerative diseases. Neurodegeneration.

[B20] Krieglstein K, Henheik P, Farkas L, Jaszai J, Galter D, Krohn K, Unsicker K (1998). Glial cell line-derived neurotrophic factor requires transforming growth factor-beta for exerting its full neurotrophic potential on peripheral and CNS neurons. J Neurosci.

[B21] Suzumura A, Sawada M, Yamamoto H, Marunouchi T (1993). Transforming growth factor-beta suppresses activation and proliferation of microglia in vitro. J Immunol.

[B22] Benveniste EN, Tang LP, Law RM (1995). Differential regulation of astrocyte TNF-alpha expression by the cytokines TGF-beta, IL-6 and IL-10. Int J Dev Neurosci.

[B23] Vodovotz Y, Geiser AG, Chesler L, Letterio JJ, Campbell A, Lucia MS, Sporn MB, Roberts AB (1996). Spontaneously increased production of nitric oxide and aberrant expression of the inducible nitric oxide synthase in vivo in the transforming growth factor beta 1 null mouse. J Exp Med.

[B24] Seltzer Z, Dubner R, Shir Y (1990). A novel behavioral model of neuropathic pain disorders produced in rats by partial sciatic nerve injury. Pain.

[B25] Livak KJ, Schmittgen TD (2001). Analysis of relative gene expression data using real-time quantitative PCR and the 2(-Delta Delta C(T)) Method. Methods.

[B26] Dunker N, Schuster N, Krieglstein K (2001). TGF-beta modulates programmed cell death in the retina of the developing chick embryo. Development.

[B27] Krieglstein K, Richter S, Farkas L, Schuster N, Dunker N, Oppenheim RW, Unsicker K (2000). Reduction of endogenous transforming growth factors beta prevents ontogenetic neuron death. Nat Neurosci.

[B28] Krieglstein K, Strelau J, Schober A, Sullivan A, Unsicker K (2002). TGF-beta and the regulation of neuron survival and death. J Physiol Paris.

[B29] Tesseur I, Wyss-Coray T (2006). A role for TGF-beta signaling in neurodegeneration: evidence from genetically engineered models. Curr Alzheimer Res.

[B30] Tesseur I, Zou K, Esposito L, Bard F, Berber E, Can JV, Lin AH, Crews L, Tremblay P, Mathews P, Mucke L, Masliah E, Wyss-Coray T (2006). Deficiency in neuronal TGF-beta signaling promotes neurodegeneration and Alzheimer's pathology. J Clin Invest.

[B31] Krieglstein K, Suter-Crazzolara C, Fischer WH, Unsicker K (1995). TGF-beta superfamily members promote survival of midbrain dopaminergic neurons and protect them against MPP+ toxicity. EMBO J.

[B32] Brionne TC, Tesseur I, Masliah E, Wyss-Coray T (2003). Loss of TGF-beta 1 leads to increased neuronal cell death and microgliosis in mouse brain. Neuron.

[B33] Tsujino H, Kondo E, Fukuoka T, Dai Y, Tokunaga A, Miki K, Yonenobu K, Ochi T, Noguchi K (2000). Activating transcription factor 3 (ATF3) induction by axotomy in sensory and motoneurons: A novel neuronal marker of nerve injury. Mol Cell Neurosci.

[B34] Kataoka K, Kanje M, Dahlin LB (2007). Induction of activating transcription factor 3 after different sciatic nerve injuries in adult rats. Scand J Plast Reconstr Surg Hand Surg.

[B35] Luo MC, Zhang DQ, Ma SW, Huang YY, Shuster SJ, Porreca F, Lai J (2005). An efficient intrathecal delivery of small interfering RNA to the spinal cord and peripheral neurons. Mol Pain.

[B36] Xiao BG, Bai XF, Zhang GX, Link H (1997). Transforming growth factor-beta1 induces apoptosis of rat microglia without relation to bcl-2 oncoprotein expression. Neurosci Lett.

[B37] Toru-Delbauffe D, Baghdassarian-Chalaye D, Gavaret JM, Courtin F, Pomerance M, Pierre M (1990). Effects of transforming growth factor beta 1 on astroglial cells in culture. J Neurochem.

[B38] Morganti-Kossmann MC, Kossmann T, Brandes ME, Mergenhagen SE, Wahl SM (1992). Autocrine and paracrine regulation of astrocyte function by transforming growth factor-beta. J Neuroimmunol.

[B39] Makwana M, Jones LL, Cuthill D, Heuer H, Bohatschek M, Hristova M, Friedrichsen S, Ormsby I, Bueringer D, Koppius A, Bauer K, Doetschman T, Raivich G (2007). Endogenous transforming growth factor beta 1 suppresses inflammation and promotes survival in adult CNS. J Neurosci.

[B40] Kawasaki Y, Zhang L, Cheng JK, Ji RR (2008). Cytokine mechanisms of central sensitization: distinct and overlapping role of interleukin-1beta, interleukin-6, and tumor necrosis factor-alpha in regulating synaptic and neuronal activity in the superficial spinal cord. J Neurosci.

[B41] Samad TA, Moore KA, Sapirstein A, Billet S, Allchorne A, Poole S, Bonventre JV, Woolf CJ (2001). Interleukin-1beta-mediated induction of Cox-2 in the CNS contributes to inflammatory pain hypersensitivity. Nature.

[B42] Ji RR, Suter MR (2007). p38 MAPK, microglial signaling, and neuropathic pain. Mol Pain.

[B43] Chaplan SR, Bach FW, Pogrel JW, Chung JM, Yaksh TL (1994). Quantitative assessment of tactile allodynia in the rat paw. J Neurosci Methods.

[B44] Dixon WJ (1980). Efficient analysis of experimental observations. Annu Rev Pharmacol Toxicol.

[B45] Hargreaves K, Dubner R, Brown F, Flores C, Joris J (1988). A new and sensitive method for measuring thermal nociception in cutaneous hyperalgesia. Pain.

[B46] Yaksh TL, Rudy TA (1976). Chronic catheterization of the spinal subarachnoid space. Physiol Behav.

[B47] Zhang J, Rivest S (1999). Distribution, regulation and colocalization of the genes encoding the EP2- and EP4-PGE2 receptors in the rat brain and neuronal responses to systemic inflammation. Eur J Neurosci.

